# Exploratory analysis of the association between body composition albumin-bound paclitaxel induced peripheral neuropathy

**DOI:** 10.3389/fphar.2026.1644444

**Published:** 2026-02-06

**Authors:** Ying Jiang, Jiayuan Guo, Juan Zhao, Feng Chen, Jixiang Hou, Xiaorong Li, Zhenhao Li, Gaowa Jin, Quanfu Li

**Affiliations:** 1 Department of Medical Oncology, Ordos Central Hospital, Ordos, Inner Mongolia, China; 2 The First Clinical Medical College of Wenzhou Medical University, Wenzhou, Zhejiang, China

**Keywords:** albumin-bound nanoparticles, antineoplastic agents, neoplasms, peripheral nervous system diseases, skeleton, diagnostic imaging

## Abstract

**Objective:**

This study aimed to examine the relationship between L3 Skeletal Muscle Index (L3SMI) and the incidence and severity of chemotherapy-induced peripheral neuropathy (CIPN) in patients with malignant tumors treated with nab-PTX (albumin-bound paclitaxel) monotherapy or in combination with cisplatin or carboplatin.

**Methods:**

This study included 52 patients with complete clinical data. The patients’ baseline demographics, disease characteristics, body composition, PG-SGA scale and chemotherapy regimens,was evaluated prior to chemotherapy initiation. Blood samples were collected within 1 hour following the final nab-PTX dose. CIPN was assessed before the third chemotherapy cycle. Patients receiving nab-PTX doses on day 1 were categorized into Group A (n = 36), while those receiving doses on days 1 and 8 were assigned to Group B (n = 16). Group A was further divided into the sarcopenia subgroup (A1) and the non-sarcopenia group (A2), while Group B was divided into the sarcopenia subgroup (B1) and the non-sarcopenia subgroup (B2). The relationship between L3SMI, CIPN incidence and severity were analyzed, and the impact of L3SMI on blood drug concentrations was also investigated.

**Results:**

The pathological types of enrolled patients included: lung cancer, esophageal carcinoma, cervical cancer, and ovarian cancer. The incidence of CIPN ≥ B grade was significantly higher in sarcopenia subgroups compare with non-sarcopenia in Group A (A1 vs. A2, *P* = 0.042). Severe CIPN (defined as ≥ grade C) showed a numerically higher occurrence in the sarcopenia subgroups compared to their non-sarcopenia counterparts across groups, although these comparisons did not reach statistical significance. Furthermore, the incidence of CIPN was significantly higher in Group A than in Group B. Among patients receiving nab-PTX doses on day 1, the average dose per kilogram of lean body mass (LBM) was significantly greater in the sarcopenia subgroup compared to the non-sarcopenia subgroup (*P* < 0.001).

**Conclusion:**

Sarcopenia significantly increases the incidence and severity of CIPN in patients undergoing nab-PTX-based chemotherapy. This association may be attributed to the effectively higher dose of nab-PTX per kilogram of lean body mass in patients with sarcopenia, despite the absence of a direct correlation between L3SMI and measured blood drug concentrations. These findings highlight the importance of body composition assessment prior to chemotherapy, as patients with sarcopenia may require enhanced monitoring for CIPN or individualized dosing considerations.

## 1 Introduction

Muscle wasting is highly prevalent among adults with cancer. The 2019 consensus from the Asian Working Group on Sarcopenia (AWGS) defines sarcopenia as the age-related loss of skeletal muscle mass, strength, and/or physical function (Chen et al., 2020). In gerontology, the use of CT scans to assess cross-sectional images at the level of the third lumbar vertebra (L3) is considered the gold standard for diagnosing sarcopenia (Kong et al., 2022). Numerous studies have shown that cancer patients with sarcopenia are more vulnerable to chemotherapy-related adverse effects (Martin et al., 2013; Caan et al., 2018; Prado et al., 2008; Shachar et al., 2016; Ryan et al., 2019). Furthermore, reduced L3 Skeletal Muscle Index (L3SMI) at the time of cancer diagnosis is linked to lower overall survival rates in patients with solid tumors (Shachar et al., 2016; Youn et al., 2021). Emori et al. (2022) identified L3SMI as an independent prognostic factor influencing both progression-free survival (PFS) and overall survival (OS) in individuals with cancer.

Chemotherapy-induced peripheral neuropathy (CIPN) is a common dose-limiting adverse effect of chemotherapeutic agents, characterized by varying degrees of sensory, motor, and/or autonomic dysfunction. Sensory symptoms are most prominent in the distal extremities, typically first manifesting in the feet and hands, following a typical glove and stocking distribution. These symptoms include tingling, numbness, loss of vibration sense, altered tactile sensation, abnormal sensations, and sensory disturbances triggered by touch or changes in temperature (cold and warm) (Park et al., 2013). In severe cases, complete loss of sensory perception may occur. Motor symptoms, less common than sensory symptoms, include distal weakness, motor impairment, and disturbances in gait and balance (Kolb et al., 2016). Although several treatment strategies for CIPN have been proposed, including ion channel-targeting therapy, anti-inflammatory therapy, and antioxidants, there is limited conclusive evidence to support the definitive prevention or treatment of CIPN (Desforges et al., 2022). Consequently, identifying high-risk factors for CIPN development remains of critical practical importance.

The nanoparticle nab-PTX (albumin-bound paclitaxel), a conjugate of albumin and paclitaxel (PTX), has demonstrated improved remission rates and longer PFS in patients with advanced metastatic breast cancer and non-small cell lung cancer, as well as extending survival in advanced pancreatic cancer. CIPN is one of the most common adverse reactions associated with nab-PTX, characterized by strong and persistent symptoms that can impact patients’ adherence to treatment (Park et al., 2013; Gradishar et al., 2005; Socinski et al., 2012; Von Hoff et al., 2013). Currently, the clinical evaluation criteria for peripheral neurotoxicity related to nab-PTX primarily rely on the Taxane Patient Neurotoxicity Questionnaire (PNQ) (Staff et al., 2017; Guo et al., 2023). Studies show that dose-limiting toxicity (DLT) is more prevalent among patients receiving a higher dose of nab-PTX per kilogram of Lean Body Mass (LBM), LBM defined as the total mass of all components in the body excluding adipose tissue, primarily comprising skeletal muscle, organs, bones, body fluids, and other non-fat tissues (Youn et al., 2021). Additionally, research has established that body composition indicators, such as L3SMI, are related to the pharmacokinetics of chemotherapeutic drugs (Prado et al., 2007; Ali et al., 2016). Previous clinical studies have confirmed that systemic drug exposure is proportional to the administered dose (Ibrahim et al., 2002). Chen et al. (2014) found that factors such as age, sex, and body surface area (BSA) do not appear to affect the elimination of paclitaxel. In a 2025 clinical study published in the Journal of Clinical Oncology (JCO), Assenat et al. (2025) demonstrated that, in the adjuvant setting for stage III colon cancer, the use of an L3SMI-based oxaliplatin dosing regimen significantly reduces oxaliplatin-induced peripheral neuropathy (OIPN) and improves quality of life without compromising relapse-free survival (RFS) or overall survival (OS). Prior research has focused on non-lipophilic drugs such as 5-Fu, gemcitabine, and epirubicin in European and American populations. Currently, nab-PTX is a lipid-soluble drug which administered based on the patient’s BSA, without consideration for individual differences in body composition. This approach may result in serious adverse reactions among cancer patients with sarcopenia who undergo nab-PTX treatment (Van Doorn et al., 2021). L3SMI is typically assessed by identifying and quantifying pixel regions within specific Hounsfield unit ranges, with cross-sectional images at the third lumbar vertebra level (Mourtzakis et al., 2008). As a representative method for analyzing body composition, L3SMI has shown that sarcopenia is associated with increased susceptibility to chemotherapy-related adverse effects in patients diagnosed with cancer (Hopkins and Sawyer, 2017).

The present study investigates whether sarcopenia diagnosed by L3SMI modulates the incidence and severity of nab-PTX-induced CIPN, and concurrently examines the triadic relationship among skeletal muscle status, drug exposure, and neurotoxicity risk. The resulting evidence is intended to inform body-composition-adapted dosing strategies that may preserve efficacy while limiting neurotoxicity.

## 2 Materials and methods

### 2.1 Research subjects

#### 2.1.1 Collection of general data

Individuals admitted to the Department of Oncology at ORDOS Central Hospital between December 2020 and December 2022 were prospectively enrolled in this study. Eligible participants were pathologically diagnosed with malignant tumors and received either monotherapy or combination chemotherapy involving albumin-bound paclitaxel for injection (100 mg/vial, produced by Jiangsu Hengrui Pharmaceuticals Co., Ltd.) or cisplatin (carboplatin).

#### 2.1.2 Inclusion criteria

① Aged 18 years or older, with a pathological diagnosis of malignant tumors, and scheduled to receive nab-PTX monotherapy or combination chemotherapy with cisplatin (carboplatin); ② Normal liver and kidney function, and normal electrocardiogram prior to chemotherapy; biochemical indices within the following ranges: white blood cells >3.5 × 10^9^/L, neutrophils >1.5 × 10^9^/L, platelets >85 × 10^9^/L, alkaline phosphatase ≤2.5 times the upper limit of normal, serum alanine aminotransferase (ALT) and aspartate aminotransferase (AST) ≤3 times the upper limit of normal, bilirubin ≤1.5 times the upper limit of normal, serum creatinine ≤1.5 times the upper limit of normal; ③ Karnofsky Performance Status (KPS) score ≥70; ④ Absence of blood-related diseases; ⑤ No contraindications to the proposed chemotherapy regimen; ⑥ No surgical or other interventions performed between the CT scan and the start of chemotherapy that could alter body composition; ⑦ Ability to undergo regular follow-up evaluations for peripheral neuropathy; ⑧ Receiving adjuvant chemotherapy or first-line chemotherapy. For second-line chemotherapy, participants must not have previously received paclitaxel or oxaliplatin, and must not have a chemotherapy interval greater than 12 months; ⑨ Eligible for venous blood sampling for laboratory tests within 24 h after chemotherapy. This study was approved by the Medical Ethics Committee of ORDOS Central Hospital (approval number: 2020-008), and all enrolled patients provided informed consent. The study was registered with the China Clinical Trial Registry (ChiCTR2000040918).

#### 2.1.3 Exclusion criteria

① Patients with allergies to nab-PTX or platinum-based drugs; ② Patients who are bedridden at the time of enrollment and unable to participate in body composition measurements; ③ Presence of concurrent severe wasting diseases (e.g., tuberculosis, hyperthyroidism, systemic lupus erythematosus, etc.); ④ Patients on long-term hormone therapy (>1 month); ⑤ Patients with concurrent conditions that affect drug excretion or metabolism, such as AIDS (Acquired Immune Deficiency Syndrome), kidney disease, or active hepatitis; ⑥ Patients who voluntarily discontinued anti-tumor treatment or failed to undergo regular follow-up for peripheral neuropathy evaluation; ⑦ Bilirubin levels exceeding 1.5 times the upper limit of normal and serum creatinine levels exceeding 1.5 times the upper limit of normal; ⑧ Patients with liver, gallbladder, or pancreatic metastases that could interfere with nab-PTX metabolism; ⑨ Patients with concurrent diabetes; ⑩ Patients with a history of peripheral neuropathy caused by chemotherapy or other factors (e.g., diabetic peripheral neuropathy, vitamin B deficiency, peripheral nerve injury, or carpal tunnel syndrome).

### 2.2 Data collection

#### 2.2.1 General information collection

Basic information was collected from all participants before the initiation of chemotherapy, including name, sex, age, place of origin, contact information, type of disease, pathological stage, initial treatment plan, and other relevant clinical information.

#### 2.2.2 Measurement of height and weight

Before each chemotherapy session, height and weight measurements were conducted following standardized methods. The Body Mass Index (BMI) and Body Surface Area (BSA) was calculated using specific formulas. Standard procedures for height and weight measurement included: ① Patients stood on the platform with a straight posture, chest out, and abdomen in. ② Heels were pressed together and toes angled outward at approximately 60°. Knees remained straight and together. ③ Patients looked straight ahead, with both eyes aligned, ensuring the lower margin of the eye sockets were level with the upper edge of the earlobes. The shoulders, buttocks, and heels made contact with the support column simultaneously, with arms hanging naturally at the sides and palms facing the thighs. ④ The measurer’s eyes aligned with the horizontal measuring plate when recording the measurements. BMI was calculated as: weight (kg)/height^2^ (m^2^); BSA was calculated using the formula: [height (cm) + weight (kg) – 60]/100 (m^2^). According to the BMI standards of the Chinese Anti-Cancer Association’s Professional Committees on Tumor Nutrition, the categories are as follows: <18.5 kg/m^2^ is underweight (malnutrition), 18.5–23.9 kg/m^2^ is normal, 24–27.9 kg/m^2^ is overweight, and ≥28 kg/m^2^ is obesity.

#### 2.2.3 Assessment with nutritional scales

Before the first cycle of chemotherapy, the following checks were performed on each patient, and the data were recorded:

① NRS 2002 and PG-SGA Assessment: All subjects underwent scale assessments conducted by a professional nutritionist within 48 h of admission. The NRS 2002 score was derived directly from the scale (Hopkins and Sawyer, 2017): An NRS score of ≥3 indicated nutritional risk, requiring intervention; an NRS score of <3 indicated no risk, though weekly screening was conducted during hospitalization. The PG-SGA included both qualitative and quantitative assessments, which were also derived directly from the scale (Mourtzakis et al., 2008): Qualitative/Quantitative Results: A/≤ 1 point (well-nourished); B/2 to 8 points (suspected or moderate malnutrition); C/≥ 9 points (severe malnutrition).

#### 2.2.4 Body composition measurement

##### 2.2.4.1 Abdominal CT scan

All research subjects underwent a lower lumbar spine CT scan at the L3 vertebral level prior to chemotherapy, with specific standards set between −29 and +150 Hounsfield Units for muscle tissue. Abdominal CT scanning using routine clinical non-contrast CT. The scan was conducted with a slice thickness of 5 mm. The analysis involved two consecutive transverse sections of the L3 vertebral body to assess the muscle area. Using the TPS (XIO) software for radiation therapy, the skeletal muscle area was calculated, including muscles such as the psoas major, quadratus lumborum, erector spinae, external abdominal oblique, internal abdominal oblique, and transverse abdominis muscles.

Research indicates that the third lumbar vertebra serves as a key landmark for body composition assessment. The ratio of skeletal muscle to adipose tissue at this level correlates with whole-body tissue proportions. By analyzing CT images at the L3 vertebra with imaging software, whole-body lean-tissue compartments can be estimated from a single cross-sectional slice with high precision (Mourtzakis et al., 2008). We therefore assumed that findings based on L3SMI are also valid for whole-body LBM.

L3SMI (cm^2^/cm^2^) was computed as

L3SMI = L3 skeletal muscle area (cm^2^)/height^2^ (cm^2^).

Whole-body LBM (kg) was then predicted from the same L3 muscle area using the regression equation derived by Mourtzakis et al. (2008) (r = 0.94, SEE = 0.72 kg):

LBM (kg) = 0.30 (kg·cm^−2^) × L3 skeletal muscle area (cm^2^) + 6.06 (kg).

The coefficient 0.30 carries the unit kg·cm^−2^ and the intercept 6.06 the unit kg, ensuring dimensional consistency. Both values are empirical regression parameters obtained in 51 cancer patients against DXA-measured LBM, not universal physical constants.

##### 2.2.4.2 Bioelectrical impedance analysis (BIA) test

All enrolled patients underwent body composition measurements (including body fat mass, skeletal muscle mass, and body fat percentage) using the Multi-frequency Bioelectrical Impedance Analyzer from the Nutrition Department of ORDOS Central Hospital (Model: DBA-550). The measurement was conducted in an environment with a suitable temperature (20 °C–25 °C), with patients fasting, with an empty bowel and bladder prior to the test. Measurements were avoided following exercise, showering, or during a woman’s menstrual period (Shi et al., 2020).

#### 2.2.5 Administration method

The specific dosing regimen was as follows: nab-PTX 260 mg/m^2^ on day 1 or 130 mg/m^2^ on days 1 and 8, and cisplatin 75 mg/m^2^ on days 1–3 (carboplatin AUC (Area Under the Curve) = 5 on day 1). The nab-PTX dosing regimen consisted of a single daily dose of 260 mg/m^2^ on day 1 or a split dose of 130 mg/m^2^ on both days 1 and 8. Nab-PTX was infused within 30 min. The start and end times of the nab-PTX infusion were accurately documented. Each 21-day period represented one chemotherapy cycle.

#### 2.2.6 Grouping criteria

In the enrolled patients, Group A was defined as nab-PTX administered as a single daily dose of 260 mg/m^2^ on day 1, and Group B was defined as the split dose given on days 1 and 8 (130 mg/m^2^ on both days). Based on the Asian L3SMI sarcopenia assessment criteria (men: L3SMI <36 cm^2^/m^2^; women: L3SMI <29 cm^2^/m^2^), Group A was subdivided into the sarcopenia subgroup A1 and the non-sarcopenia subgroup A2, while Group B was subdivided into the sarcopenia subgroup B1 and the non-sarcopenia subgroup B2 (Iritani et al., 2015).

#### 2.2.7 Laboratory procedures

After nab-PTX administration, 2 mL of plasma was collected 24 h later and preserved. The start time and end time of the nab-PTX infusion, specific medication used, and dosage was accurately recorded. Additionally, 2 mL of peripheral whole blood was collected within 24 h of the infusion start, using a K2EDTA anticoagulated collection tube. The blood sample was placed on ice or stored in a refrigerator at 2 °C–8 °C within 10 min of collection. Plasma was separated from the blood through centrifugation within 12 h after collection, and stored in the laboratory’s −80 °C freezer.

#### 2.2.8 CIPN measurement

Peripheral neuropathy assessment was performed for enrolled patients before the start of the third chemotherapy cycle involving nab-PTX, according to the Taxanes PNQ. The grading for CIPN was as follows: A: None; B: Slight numbness; C: Moderate numbness, mild impact on daily life; D: Moderate to severe numbness, moderate impact on daily life; E: Severe numbness, affecting most normal daily activities (Staff et al., 2017). Severe CIPN was defined as peripheral neurotoxicity of grade C or higher based on the criteria from the study by Guo et al. (2023).

#### 2.2.9 Sample testing and data analysis

The collected blood samples were sent to the Laboratory of Sun Yat-sen University Cancer Center, where the blood drug concentration of nab-PTX was measured using a dedicated testing kit. Relevant analyses were conducted based on the blood drug concentration results.

### 2.3 Statistical methods

Data analysis was performed using IBM SPSS 26.0 and GraphPad Prism 8.0 statistical software. For data that follow a normal distribution, measurement data were presented as the mean ± standard deviation (‾x ± s). Independent sample t-tests or approximate t-tests were used for univariate analysis of factors such as SMI and LBM. For measurement data that do not follow a normal distribution, they were presented as the median and interquartile range [M(QL, QU)], with intergroup comparisons made using the Mann-Whitney (U) test for independent samples. Count data were expressed as rates (%), with intergroup comparisons made using Fisher’s exact probability method or the Chi-square (X^2^) test. A value of *P* < 0.05 was considered statistically significant.

## 3 Results

### 3.1 Results of general data analysis

The study included 52 patients with complete clinical data, consisting of 36 patients in Group A (receiving nab-PTX on a day 1) and 16 patients in Group B (receiving nab-PTX on days 1 and 8). All patients were diagnosed with malignant tumors in cluding lung cancer, esophageal carcinoma, cervical cancer and ovarian cancer undergoing nab-PTX chemotherapy. The general clinical characteristics, body composition, chemotherapy regimen, and disease classification of the patients were recorded. The results indicated that the mean age of Group A was 61.58 ± 9.10 years, and the mean age of Group B was 60.83 ± 15.27 years, with ages ranging from 37 to 79 years. Significant differences in L3SMA (L3 Skeletal Muscle Area) and LBM were observed between subgroups A1 VS A2 and B1 VS B2 (*P* < 0.05) categorized by CT scans, the muscle data measured by BIA showed no statistically significant difference between the two groups, which is consistent with previous studies (Guo et al., 2023) (see Tables 1 and 2).

**TABLE 1:** Characteristics of patients in the single-day medication group (Group A, N = 36), presented as (‾x ± s) or n (%).

General characteristics	Sarcopenia group A1 (N = 20)	Non-sarcopenia group A2 (N = 16)	​
Gender			
Male	15 (75.00%)	9 (56.25%)	0.298
Female	5 (25.00%)	7 (43.75%)	
Patient characteristics			
Age (years)	64.20 ± 7.16	58.31 ± 10.38	0.052
Height (cm)	168.40 ± 8.60	166.69 ± 7.25	0.529
Weight (kg)	58.09 ± 9.58	64.21 ± 11.00	0.083
Body mass index (kg/m^2^)	20.42 ± 2.60	23.01 ± 3.60	0.017*
<18.5 low body weight	6 (30.00%)	2 (12.50%)	0.059
18.5–23.9 normal	12 (60.00%)	7 (43.75%)	​
24.0–27.9 overweight	2 (10.00%)	7 (43.75%)	​
≥28.0 obesity	0 (0%)	0 (0%)	​
BSA (m^2^)	1.66 ± 0.16	1.71 ± 0.16	0.413
Muscle mass (kg)	42.13 ± 6.97	45.92 ± 8.87	0.160
Lean body mass (kg)	45.59 ± 7.58	49.62 ± 9.57	0.168
Fat percentage	0.22 ± 0.08	0.22 ± 0.09	0.897
FFMI (kg/m^2^)	16.28 ± 2.12	17.87 ± 2.36	0.042*
L3SMA (cm^2^)	81.92 ± 15.99	109.89 ± 23.70	0.000*
L3SMI (cm^2^/m^2^)	28.75 ± 4.77	39.24 ± 6.42	0.000*
LBM (kg)	30.64 ± 4.80	39.03 ± 7.11	0.000*
PG-SGA scale			
≤1 score	0 (0%)	1 (6.25%)	0.142
2–8 scores	14 (70.00%)	14 (87.50%)	
≥9 scores	6 (30.00%)	1 (6.25%)	
Primary cancer site			
Lung cancer	11 (55.00%)	6 (37.50%)	0.312
Cervical cancer	0 (0%)	2 (12.50%)	
Ovarian cancer	2 (10.00%)	2 (12.50%)	
Esophageal carcinoma	4 (20.00%)	1 (6.25%)	
Other	3 (15.00%)	5 (31.25%)	
Treatment options			
nab-PTX monotherapy	4 (20.00%)	6 (37.50%)	0.285
nab-PTX in combination with cisplatin/carboplatin	16 (80.00%)	10 (62.50%)	

* indicates *P* < 0.05, there is a statistically significant difference.

**TABLE 2:** Characteristics of the B group patients on day 1 and day 8 (N = 16), presented as (‾x ± s) or n (%).

General characteristics	Sarcopenia group B1 (N = 3)	Non-sarcopenia group B2 (N = 13)	*P*
Gender			
Male	2 (66.67%)	10 (76.92%)	1.00
Female	1 (33.33%)	3 (23.08%)	
Patient characteristics			
Age (years)	61.67 ± 10.21	64.46 ± 9.22	0.695
Height (cm)	166.00 ± 5.29	169.54 ± 4.59	0.259
Weight (kg)	58.30 ± 3.56	68.67 ± 10.25	0.113
Body mass index (kg/m^2^)	21.19 ± 1.62	23.84 ± 4.06	0.294
<18.5 low body weight	0 (0%)	1 (7.69%)	0.614
18.5–23.9 normal	3 (100.00%)	6 (46.15%)	​
24.0–27.9 overweight	0 (0%)	3 (23.08%)	​
≥28.0 obesity	0 (0%)	3 (23.08%)	​
BSA (m^2^)	1.64 ± 0.07	1.78 ± 0.11	0.053
Muscle mass (kg)	41.37 ± 5.75	47.74 ± 5.10	0.183
Lean body mass (kg)	44.80 ± 6.26	51.61 ± 5.53	0.168
Fat percentage	0.23 ± 0.09	0.24 ± 0.09	0.886
FFMI (kg/m^2^)	17.48 ± 3.47	18.45 ± 3.20	0.649
L3SMA (cm^2^)	83.91 ± 23.10	121.26 ± 20.51	0.014*
L3SMI (cm^2^/m^2^)	30.15 ± 6.72	42.17 ± 6.86	0.068
LBM (kg)	31.23 ± 6.93	42.44 ± 6.15	0.014*
PG-SGA scale			
≤1 score	0 (0%)	1 (7.69%)	1.000
2–8 scores	2 (66.67%)	9 (69.23%)	
≥9 scores	1 (33.33%)	3 (23.08%)	
Primary cancer site			
Lung cancer	2 (66.67%)	4 (30.77%)	0.791
Cervical cancer	0 (0%)	1 (7.69%)	
Ovarian cancer	0 (0%)	1 (7.69%)	
Esophageal carcinoma	0 (0%)	3 (23.08%)	
Other	1 (33.33%)	4 (30.77%)	
Treatment options			
nab-PTX monotherapy	1 (66.67%)	3 (23.08%)	1.000
nab-PTX in combination with cisplatin/carboplatin	2 (33.33%)	10 (76.92%)	

* indicates *P* < 0.05, indicating a statistically significant difference.

### 3.2 Relationship between sarcopenia and nab-PTX-induced CIPN

The results indicated that the overall incidence of CIPN was 57.69% (30 out of 52). In Group A (single-day medication), the incidence of CIPN at various levels (≥ B grade) was significantly higher in the group with sarcopenia compared to the group without sarcopenia (A1 group vs. A2 group: 75.00% vs. 62.50%, *P* = 0.042), with a statistically significant difference. The overall incidence of CIPN (≥ B grade) in Group B (medication on days 1 and 8) was 31.25% (5/16). detailed subgroup data are provided in Table 3. The results also indicated that in both Group A (single-day medication) and Group B (medication on days 1 and 8), patients with sarcopenia the exhibited a higher incidence of severe CIPN (≥ C grade) compared to the group without sarcopenia. Although the numeric incidence of severe CIPN was higher in sarcopenia subgroups, between-group differences did not reach statistical significance (see Table 4).

**TABLE 3:** Comparison of L3SMI between patients in groups A and B and corresponding levels of CIPN.

CIPN grading	Single-day medication, group A (N = 36)		1st and 8th day medication, group B (N = 16)	
	A1 (N = 20)	A2 (N = 16)	B1 (N = 3)	B2 (N = 13)
A Grade	5 (25.00%)	6 (37.50%)	1 (33.33%)	10 (76.92%)
B Grade	7 (35.00%)	8 (50.00%)	1 (33.33%)	2 (15.39%)
C Grade	7 (35.00%)	0 (0%)	1 (33.33%)	0 (0%)
D Grade	1 (5.00%)	2 (12.50%)	0 (0%)	1 (7.69%)
E Grade	0 (0%)	0 (0%)	0 (0%)	0 (0%)
Fisher	3.351		1.465	
*P*	0.042^*^		0.214	

* indicates *P* < 0.05, indicating a statistically significant difference.

**TABLE 4:** Comparison of L3SMI and severe CIPN in patients across groups A and B.

CIPN grade	Single-day medication, group A (N = 36)		1st and 8th day medication, group B (N = 16)	
	A1 (N = 20)	A2 (N = 16)	B1 (N = 3)	B2 (N = 13)
Severe CIPN (≥C Grade/N cases, %)	8/20 (40.00%)	2/16 (12.50%)	1/3	1/13 (7.70%)
Fisher	3.351		1.465	
*P*	0.133		0.350	

*indicates *P* < 0.05, indicating a statistically significant difference.

*Comparisons involving subgroups with n < 5 are not presented as percentages to avoid over-interpretation.

We also further analyzed the relationship between administration method and nab-PTX-induced CIPN,the results indicate that patients receiving single-day medication had a significantly higher likelihood of developing CIPN at various levels (≥ B grade) compared to those receiving medication on days 1 and 8 (69.44% vs. 31.25%, *P* = 0.010, see Table 5). The comparison between the two groups indicated a statistically significant difference.

**TABLE 5:** Comparison of CIPN incidence between groups A and B.

Group	CIPN (≥B Grade/N cases, %)	χ^2^	*P*
Single-day medication, group A (N = 36)	25/36 (69.44%)	6.62	0.010^*^
1st and 8th day medication, Group B (N = 16)	5/16 (31.25%)		

* indicates P < 0.05, indicating a statistically significant difference.

### 3.3 Relationship between sarcopenia and nab-PTX dose per kilogram of LBM in patients treated with single-day medication

**FIGURE 1:**
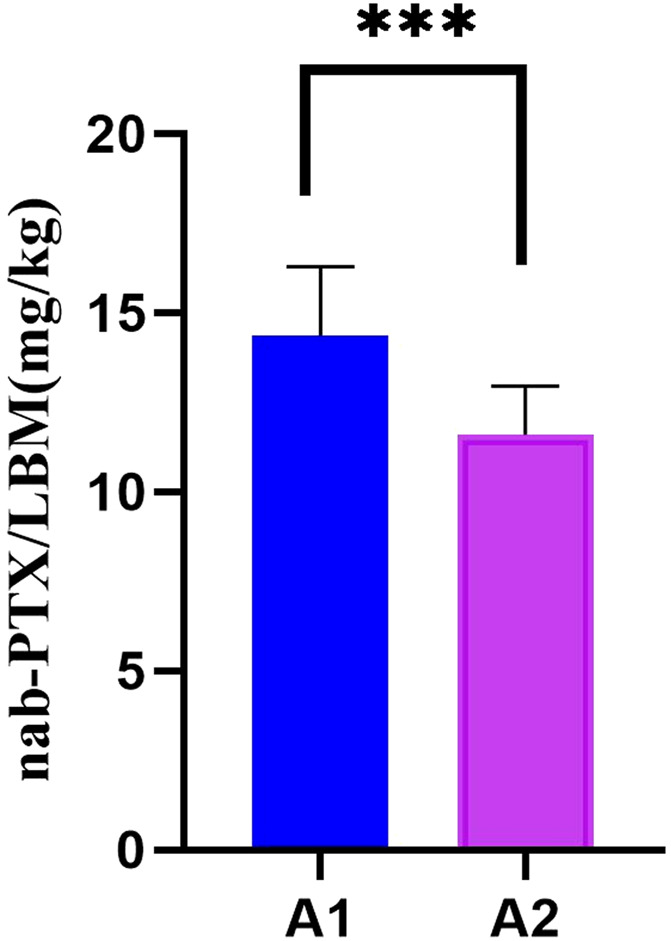
The correlation between nab-PTX dosage per kilogram of LBM and sarcopenia. Note: *** indicates P < 0.001, indicating a statistically significant difference. Adapted from Li Z, et al. (2024), originally published in Frontiers in Oncology under CC-BY license.

**FIGURE 2:**
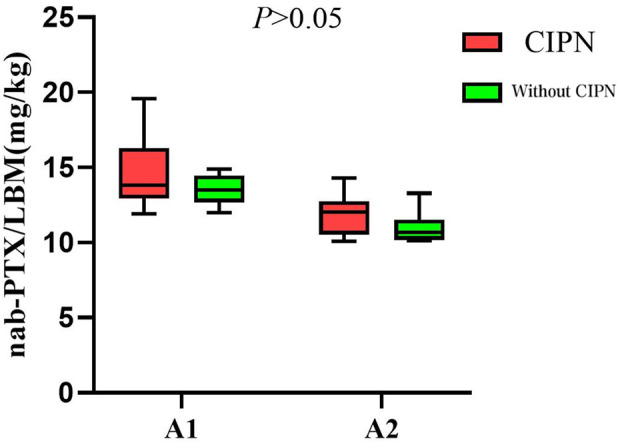
The correlation between nab-PTX dosage per kilogram of LBM and the incidence of CIPN in groups with sarcopenia.

**FIGURE 3:**
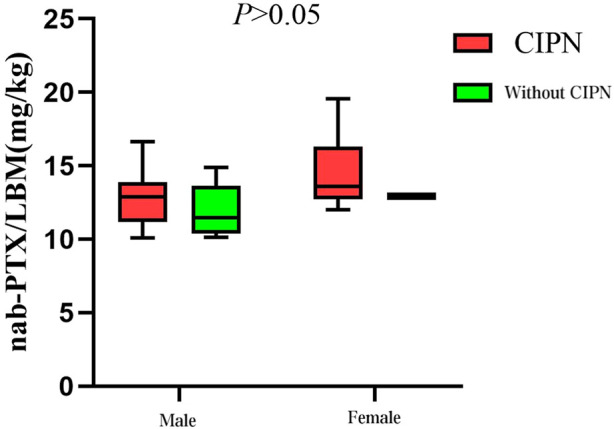
The correlation between nab-PTX dosage per kilogram of LBM and the incidence of CIPN across gender groups.

In patients receiving single-day nab-PTX administration (Group A), those with sarcopenia received a significantly higher average dose per kilogram of LBM compared to non-sarcopenia patients (14.37 vs. 11.60 mg/kg LBM, P < 0.001; Figure 1). Further exploratory subgroup analyses examined the association between LBM-adjusted nab-PTX dose and the occurrence of CIPN (≥ grade B). A consistent, though statistically non-significant, numerical trend was observed across groups: patients who developed CIPN tended to receive a higher average dose per kg LBM compared to those without CIPN. This trend was noted within both the sarcopenia (14.63 vs. 13.74 mg/kg LBM) and non-sarcopenia subgroups (11.97 vs. 10.99 mg/kg LBM), as well as in sex-stratified analyses (Males: 12.84 vs. 12.04 mg/kg LBM; Females: 14.49 vs. 13.28 mg/kg LBM) (Figures 2, 3). It should be noted that the sample sizes for some of these subgroups were limited, which precludes definitive conclusions.

### 3.4 Relationship between nab-PTX blood drug concentrations, sarcopenia, and gender in patients treated with single-day medication

In Group A (single-day nab-PTX administration), nab-PTX blood drug concentrations were analyzed based on sarcopenia status and gender (see Table 6). The results indicated no statistically significant differences in nab-PTX blood drug concentrations between patients with sarcopenia and those without sarcopenia (*P* > 0.05) (see Table 6). Similarly, no significant differences were observed in drug concentrations between male and female patients (*P* > 0.05).

**TABLE 6:** Comparison of sarcopenia and gender with nab-PTX blood drug concentrations in group A.

Blood concentration	Single-day medication, group A (N = 36)		Gender (N = 36)	
	A1 (N = 20)	A2 (N = 16)	Male (N = 24)	Female (N = 12)
nab-PTX concentration (ng/mL)	55.55 (40.70, 66.90)	66.45 (47.70, 79.70)	55.55 (40.70, 62.60)	71.00 (47.70, 86.10)
Mann-Whitney (U)	126.50		92.00	
*P*	0.290		0.081	

## 4 Discussion

In both the single-day group (Group A) and split-dose group (Group B) administration regimens, patients with sarcopenia exhibited higher incidences of CIPN, particularly severe CIPN (≥ C grade), compared to the group without sarcopenia, indicating that sarcopenia may increase the incidence of CIPN induced by nab-PTX. The overall incidence of severe CIPN (≥ C grade) was 27.78% (10 out of 36) among patients receiving single-day nab-PTX treatment. These results align with previous studies reporting a higher incidence of chemotherapy-related toxicities in patients with sarcopenia. Shachar et al. (2017) observed that patients with metastatic breast cancer and sarcopenia treated with docetaxel were more likely to experience DLT, although their study included various chemotherapy drugs such as PTX, nab-PTX, and docetaxel with inconsistent doses and a broader toxicity evaluation framework. Barret et al. (2014) conducted a prospective multi-center study, identifying sarcopenia as the sole factor associated with grade 3 to 4 chemotherapy toxicity in 51 patients with metastatic colorectal cancer (OR = 13.55, *P* = 0.043). Prado et al. (2009) found similar results, highlighting sarcopenia as a key predictor of chemotherapy toxicity and progression-free survival in women with metastatic breast cancer treated with capecitabine (*P* = 0.03, *P* = 0.04). Compared to patients without sarcopenia, those with sarcopenia had a threefold higher risk of chemotherapy toxicity (Dijksterhuis et al., 2019). Kurita et al. (2019) evaluated 82 patients with pancreatic cancer receiving FOLFIRINOX (oxaliplatin, calcium folinate, irinotecan, fluorouracil) and identified sarcopenia as significantly associated with hematological toxicity (*P* = 0.008). Dijksterhuis et al. (2019) studied 88 patients with advanced esophageal or gastric cancer receiving standard first-line palliative treatment and found that pretreatment sarcopenic obesity was independently linked to post-chemotherapy grade 2 to 4 neuropathy (OR: 3.82, 95% CI: 1.20–12.18).

However, the findings of this study differ from those of Freckelton et al. (2019), who examined nab-PTX treatment in advanced pancreatobiliary cancer and reported no correlation between L3SMI and chemotherapy toxicity. This discrepancy may be due to differences in the toxicity evaluation criteria, which included a broader range of adverse reactions, and the fact that the assessment was limited to the first chemotherapy cycle. Furthermore, many studies have used objective criteria such as DLT or hematological toxicity to evaluate chemotherapy toxicity. To our knowledge, this is the first study to study the relationship between sarcopenia and peripheral neuropathy induced by nab-PTX chemotherapy in a northern Chinese population. The findings suggest that sarcopenia is likely a risk factor for the development of peripheral neuropathy following nab-PTX treatment.

This study provides further insight into one possible explanation for the higher incidence of CIPN in patients with sarcopenia: the higher doses of nab-PTX per kilogram of LBM they receive. Given the smaller sample size in the single-day treatment groups (on days 1 and 8), the analysis focused on the single-day treatment group, where a notable difference in the dose of nab-PTX per kilogram of LBM was observed. In both the sarcopenia and gender groups, the mean dose of nab-PTX per kilogram of LBM was higher in patients who developed CIPN compared to those without CIPN. Particularly, the group with sarcopenia received a significantly higher mean dose of nab-PTX per kilogram of LBM compared to the group without sarcopenia (14.37 mg/kg LBM vs. 11.60 mg/kg LBM, *P* < 0.001). This suggests that patients with sarcopenia, who receive higher doses of nab-PTX per kilogram of LBM, are at an increased risk of developing CIPN.

These findings align with a study by Youn et al. (2021), which examined 152 patients treated with first-line gemcitabine plus nab-PTX. In their study, patients receiving higher doses of nab-PTX per kilogram of LBM (ranging from 0.98 to 8.76 mg/kg LBM) had a significantly higher incidence of DLT and peripheral neuropathy (58.10% vs. 36.40%, *P* < 0.05). Similarly, Ali et al. (2016) found that among colon cancer patients receiving the FOLFOX chemotherapy regimen, 39.9% of patients in the highest dose per kilogram of LBM group experienced DLT, with 25% developing neuropathy, compared to only 8.3% in the lowest dose group, with no neuropathy cases. These differences in DLT and neuropathy incidence were statistically significant (*P* < 0.05). Additionally, in this study, 11 out of 12 female patients receiving single-day treatment experienced various grades of peripheral neurotoxicity, which is consistent with the known differences L3SMI between men and women. Female patients, with relatively lower LBM, tend to experience a higher intensity of nab-PTX treatment (Youn et al., 2021).

The findings of this study are consistent with clinical observations. It was found that patients receiving nab-PTX on days 1 and 8 had a significantly lower incidence of CIPN compared to those receiving single-day treatment (31.25% vs. 69.44%, *P* = 0.010). Additionally, in the single-day treatment group, the mean age of the group with sarcopenia was significantly higher than that of the non-sarcopenic group (64.20 ± 7.16 years vs. 58.31 ± 10.38 years, *P* = 0.052), which reflects the well-established correlation between aging and increased prevalence of sarcopenia (Hertz et al., 2022). Traditional BSA dosing methods do not adequately account for changes in body composition due to aging, inadequate nutritional intake, and increased catabolism in patients with cancer. On the basis of previously study, the use of an LBM-based oxaliplatin dosing regimen significantly reduces oxaliplatin-induced peripheral neuropathy (OIPN) and improves quality of life without compromising relapse-free survival (RFS) or overall survival (OS) in the adjuvant setting for stage III colon cancer (Ali et al., 2016; Assenat et al., 2025). Therefore, it would become essential to assess the impact of L3SMI on the adverse effects of chemotherapy to better understand its relationship with chemotoxicity. This can help develop individualized dosing strategies that preserve efficacy while minimizing toxicity. Moreover, our findings suggest that fractionated infusion methods could be beneficial for older adults with sarcopenia and poor nutritional status, helping reduce the incidence of CIPN and improving treatment adherence.

**FIGURE 4:**
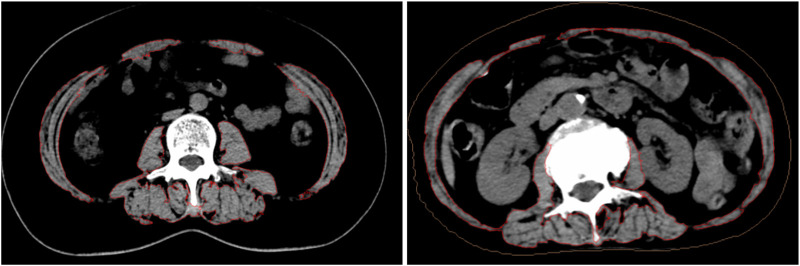
BSA remains same while L3SMA varies.

The study also used the Asian L3SMI sarcopenia assessment criteria, which identified sarcopenia in 44.23% of the 52 enrolled patients (23 out of 52) (Chen et al., 2020; Kong et al., 2022; Guo et al., 2023; Iritani et al., 2015). In contrast, when applying the widely used Canadian L3SMI criteria proposed by Martin et al. (2013) (male <43.0 cm^2^/m^2^, female <41.0 cm^2^/m^2^), the incidence of sarcopenia among the same group of patients increased to 84.62% (44 out of 52), with 15 out of 16 female patients diagnosed with sarcopenia. This discrepancy highlights the importance of considering individualized regional and ethnic differences when diagnosing sarcopenia using the L3SMI standard. Defining reference values for low skeletal muscle index at the L3 vertebra level based on computed tomography of 700 younger healthy adults datas of 1787 healthy people in four cities in northern China, using the mean minus two standard deviations protocol the reference values were 37.9 cm^2^/m^2^ and 28.6 cm^2^/m^2^ in men and women which were highly according with the reference we recommend 36 cm^2^/m^2^ and 29 cm^2^/m^2^ in men and women (Chen et al., 2020; Kong et al., 2022; Guo et al., 2023; Iritani et al., 2015). Furthermore, survival analysis based on this threshold is necessary to better determine its clinical utility in future. The high variability of L3SMI values in cancer patients with similar BSA further supports the need for personalized diagnostic approaches. In the single-day treatment group, no statistically significant difference in BSA was found between the groups with and without sarcopenia (*P* > 0.05). Figure 4 demonstrates a typical case where female patients with the same BSA exhibited an L3SMA variation by a factor of 1.5. Additionally, body composition analysis using BIA was performed, revealing no statistically significant difference in lean body mass between the groups with and without sarcopenia (*P* > 0.05). However, L3SMI showed a significant difference (*P* < 0.001), suggesting that L3SMI is a more sensitive tool for diagnosing sarcopenia than BIA. Furthermore, the primary evaluation of CIPN was conducted using a specialized assessment scale that prospectively assessed patients before their third chemotherapy cycle, providing a more accurate evaluation of CIPN incidence and severity (Iritani et al., 2015).

In this prospective observational study, the relationship between the actual blood drug concentration of nab-PTX, sarcopenia, and gender in patients receiving single-day treatment was explored. The data indicated no significant differences in the actual blood drug concentration of nab-PTX between the sarcopenia and gender groups. This finding contrasts with the results from the LBM standardization analysis, where differences were observed. Additionally, no significant correlation was found between L3SMI and actual blood drug concentration, which aligns with the study by Williams et al. Their study found no significant differences in the first-cycle 5-FU AUC (Area Under the Curve) between patients with and without sarcopenia (17.30 vs. 19.30 AUC, *P* = 0.43), nor did they observe any correlation between sarcopenia and 5-FU AUC (Williams et al., 2018).

It is important to note that these findings differ from those of Hertz DL’s study, which adjusted PTX dosing based on pharmacokinetics (PK). Hertz DL’s study indicated that sarcopenia increases the maximum concentration of PTX, and extending the infusion time to 2 or 3 h might reduce CIPN in patients with sarcopenia. Similarly, the study by Joerger et al. (2016) demonstrated that PK-guided PTX administration can reduce PTX-related neuropathy. These discrepancies may be attributed to the limited sample size and only 24 h points blood sample in our study.

Although our results do not confirm the impact of L3SMI on nab-PTX blood drug concentrations, further investigation into this relationship is warranted. Growing evidence suggests that LBM dosing may be preferable to conventional BSA dosing, particularly for drugs that are distributed and metabolized in lean tissue (Emori et al., 2022; Prado et al., 2007).

This study underscores the importance of evaluating body composition in patients with malignant tumors. It highlights the potential benefits of early screening for sarcopenia in older adults and suggests that promptly adjusting the dosing regimen could help alleviate chemotherapy-related adverse reactions. Quality of life is a primary consideration for patients undergoing palliative chemotherapy, and future research should focus on the impact of body composition-based dosing strategies on the outcomes of these patients. Achieving a balance between minimizing toxicity, maintaining therapeutic efficacy, and alleviating cancer-related symptoms is crucial. Future studies are needed to explore the interaction between skeletal muscle, drug metabolism, and clearance for nab-PTX. This would provide essential clinical evidence for developing individualized dosing regimens of nab-PTX potentially optimizing treatment outcomes and improving the quality of life for patients.

The current study has some limitations:

1. The COVID-19 pandemic, along with the exclusion of 16 patients who did not complete the BIA or blood sampling, resulted in a smaller sample size. This limitation hindered the ability to establish a critical threshold for the decrease in LBM and its impact on nab-PTX blood drug concentration levels.
2. The study methodology focused solely on measuring the muscle area at the L3 vertebral level, without analyzing or comparing the subcutaneous and visceral fat contents at this level. These factors could potentially influence nab-PTX blood drug concentration levels and chemotherapy toxicity.
3. Although this was a prospective observational study, it included patients receiving medication on different schedules (single day vs. 1st and 8th days), and patients at different cancer stages. Enrolled patients received either single-agent nab-paclitaxel or platinum-based combination chemotherapy. The imbalanced distribution between different treatment groups, as well as between the sarcopenia and non-sarcopenia groups, represents one of the limitations of this study. Therefore, a more specific analysis for each cancer stage could not be conducted. Future studies should aim to include a larger and more homogenous sample size in each group to allow for more detailed analysis.

## 5 Conclusion

1. The study confirms that patients with cancer and sarcopenia undergoing nab-PTX chemotherapy experience a higher incidence of CIPN, including severe cases. The increased incidence of CIPN can be attributed to the higher dose of nab-PTX administered per kilogram of LBM.
2. No significant correlation was found between L3SMI, gender, and the actual blood drug concentration of nab-PTX.

## Data Availability

The original contributions presented in the study are included in the article/supplementary material, further inquiries can be directed to the corresponding authors.
